# 肿瘤患者临床试验接受意愿及相关原因分析

**DOI:** 10.3779/j.issn.1009-3419.2020.01.07

**Published:** 2020-01-20

**Authors:** 慧瑶 黄, 琦 樊, 虹 房, 大维 吴, 书航 王, 颖 白, 安琪 于, 辉 王, 超 孙, 悦 俞, 元 方, 晟 杨, 菊芳 石, 瑞仙 何, 宁 李

**Affiliations:** 100021 北京，国家癌症中心/国家肿瘤临床医学研究中心/中国医学科学院北京协和医学院肿瘤医院 Department of Clinical Trials Center, National Cancer Center/National Clinical Research Center for Cancer/Cancer Hospital, Chinese Academy of Medical Sciences and Peking Union Medical College, Beijing 100021, China

**Keywords:** 肿瘤, 临床试验, 接受度, 参与意愿, Cancer, Clinical trial, Acceptance, Willingness-to-participate

## Abstract

**背景与目的:**

我国的抗肿瘤新药临床试验开展如火如荼，患者的临床试验接受度是影响临床试验开展速度和质量的重要因素。既往研究仅针对未参与过临床试验的肿瘤患者接受度展开调查，未分析参加过临床试验患者的相关情况。本研究调查并比较参加过和未参与过试验的肿瘤患者对于临床试验的接受度，并分析相关原因。

**方法:**

2018年6月-2019年4月，采用标准化问卷在中国医学科学院肿瘤医院针对肿瘤患者（参加过*vs*未参加过试验）开展调查，分析比较两组患者临床试验的接受度和差异，并分析主要原因及医生对其接受度的影响。

**结果:**

共纳入538例患者，男性51.1%，平均年龄53.5岁，43.3%的患者参加过试验。总体而言，502例（93.3%）患者愿意参加或推荐亲友参加试验，参加过试验的患者接受度较高（96.6% *vs* 90.8%, *P*=0.008）。参加过和未参加过试验患者愿意的最主要原因均为“期待最佳治疗效果”（100.0% *vs* 99.3%），次要原因分别为“可减轻经济负担”（56.0%）和“主治医生建议”（43.7%）。参加过试验的患者不愿意参加的主要原因为“放弃其他治疗选择”、“分到对照治疗组”或“额外访视影响生活”；未参加过试验的患者为“治疗效果不佳”或“出现严重不良反应”。对参加过试验的患者，医生推荐对88%患者参与试验的决策起到关键作用；对未参加过试验的患者，医生推荐可使60.9%无参与意愿者改变其选择。研究也报告了患者对临床试验获取信息和途径等的倾向选择。

**结论:**

肿瘤患者临床试验接受度普遍较高，尤其是参加过试验的患者。充分发挥主治医生的作用对提高我国肿瘤患者临床试验接受度有重要意义。

## Abstract (en)

**Background and objective:**

The clinical trials of new anti-tumor drugs are prospering in China. The acceptance of clinical trials in patients is an important factor affecting the speed and quality of clinical trials. Previous studies have investigated the acceptance of clinical trials in those cancer patients, who have never participated in a trial. This study is designed to investigate and compare the acceptance and related causes of clinical trials in cancer patients who have once participated in a clinical trial or not.

**Methods:**

From June 2018 to April 2019, a standardized questionnaire-based survey was conducted among two groups of cancer patients classified by history of clinical trial participation in Cancer hospital, Chinese Academy of Medical Science, mainly focusing on their overall acceptance of clinical trials and related considerations, including the role of attending doctors, as well as group differences between the two participants.

**Results:**

A total of 538 patients were enrolled with an average age of 53.5 years old, 51.1% of whom were males, and 43.3% of whom have never participated in a clinical trial. Overall, 502 patients (93.3%) were willing to or recommend their relatives or friends to participate in clinical trials, and patients with history of clinical trial participation had higher willingness (96.6% *vs* 90.8%, *P*=0.008). Patients were most likely to be motivated by expectation of optimal treatment (100.0% *vs* 99.3%) for both those who had once participated in a clinical trial or those not, respectively followed by financial burden reduction (56.0%) and recommendation by attending doctor (43.7%). The main reasons for unwillingness-to-participate for those who had once participated in a clinical trial were abandoning other treatment options, divided into control group or additional visits, while for those who had never participated in a clinical trial, ineffective treatment or serious adverse reactions were their main concerns. In the decision-making of clinical trial participation, 88% patients highly valued the role of recommendation by attending doctors. Among patients without trial participation history, 60.9% of those had no unwillingness-to-participate expressed that recommendation by attending doctors would change their decisions. The study also reported patients' preferences for information and access to clinical trials.

**Conclusion:**

The acceptance of clinical trials in cancer patients in our hospital is generally high, especially in patients who had a history of trial participation. It's of substantial significance to give full play to the role of doctors in improving the acceptance of clinical trials of cancer patients in China.

临床试验是新药由实验室研发走向临床运用的必经之路，肿瘤新药研究更是全球医药企业关注的重点领域，占全球新药试验的22.1%^[1]^。随着中国医药研发生态系统的改善、医院机构能力的大幅提升以及研发经验的不断增长，过去十年中国抗肿瘤临床试验数量以高速增长，年均增长达33.4%^[2, 3]^。尽管我国具有全球最大的肿瘤试验潜在受试者群体^[4]^，但患者接受度是保障我国抗肿瘤药物试验项目未来能长期可持续增长的关键指标。随着肿瘤标志物在药物试验中的广泛运用，患者是否愿意贡献组织标本/血液用于研究开展也至关重要。

既往已有三项单中心研究针对我国肿瘤患者的临床试验接受度开展相关调查^[5-7]^。由于受医院治疗水平、临床试验管理、研究医生或患者建议等多方面影响，不同医院患者临床试验参与意愿差异较大，另外既往研究存在以下问题：①调查对象仅涉及未参加过试验的患者，缺乏与参加过试验患者的结果比较；②未涉及患者对组织标本/血液的贡献意愿、对临床试验信息公布内容和途径等数据报告。

为促进我国临床试验患者管理及入组速度的提升，也为制定提高我国肿瘤患者临床试验接受意愿的策略提供科学依据，本研究将采用标准化问卷，针对中国医学科学院肿瘤医院参加过和未参加过试验的肿瘤患者开展调查，比较两组患者对临床试验的接受度，并分析主要原因及医生对其接受度的影响。

## 1 对象和方法

### 1.1 研究对象

从2018年4月-2018年12月，针对正在或曾参加中国医学科学院肿瘤医院临床试验的恶性肿瘤患者及其家属开展问卷调查。纳入标准：①明确诊断的恶性肿瘤患者或家属；②年龄≥18岁；③意识清楚，听力正常，无精神心理疾病；④签署知情同意书。排除诊断不明确，或者良性肿瘤患者及家属。本研究选取了肿瘤患者，包括：①既往参加过或正在参与试验的患者和②未参加过试验的患者作为研究对象，所有对象均签署了知情同意书。研究通过了中国医学科学院肿瘤医院伦理委员会的审查（批准批号：18-028/1629）

### 1.2 研究方法

本研究通过采用统一编制、经过专家论证及预调查的问卷展开。主要调查内容包括基本信息、临床信息、认知情况、选择倾向和意愿等。通过方便抽样针对不同组别对象来源人群进行调查，调查有人员均为经过统一培训的临床协调员。调查方式以面对面访谈调查为主，对于部分试验期已结束的患者可采用电话访谈调查。详细调查内容、调查流程及质控措施请参考同期研究方法学部分^[8]^。

### 1.3 统计学分析

数据库建立及单人双录入采用Epidata 3.1软件，逻辑核查及统计分析采用SAS 9.4。满足以下任何一条的予以剔除：①年龄缺失；②性别缺失；③是否参加过试验；④临床试验整体看法缺失；⑤参加或推荐亲友参加临床试验的意愿缺失。

本研究评价指标包括：①参加或推荐亲友参加临床试验的意愿；②贡献剩余血液/组织标本用于研究的意愿；③愿意参加或推荐亲友参加临床试验的原因和不愿意的原因；④主治医生对患者试验接受度的影响；⑤患者对临床试验信息内容及获取途径的选择倾向。年龄为计量资料，且不服从正态分布，统计描述采用均数±标准差（Mean±SD），统计分析采用秩和检验；其余变量均为计数资料，统计描述采用频数（%），统计分析采用卡方检验。所有统计检验以*P* < 0.05为差异有统计学意义。

## 2 结果

### 2.1 基本信息

本研究共纳入538例患者，男性51.1%，平均年龄53.5岁，其中233例（43.3%）参加过临床试验。总体而言，纳入对象教育程度在大学及以上占20.7%，职业以企事业/公务员/公司职员为主（34.6%）；36.1%的患者有医疗行业亲友，61.5%的患者对医务人员印象很好，26.4%的患者自报在健康节目方面经常关注。对比未参加过和参加过临床试验两组患者基本信息，结果显示年龄、性别、文化程度和医疗行业亲友状态无统计学差异，两组患者在职业（*P*=0.016）、对健康节目的关注度（*P*=0.001）和对医务人员印象（*P*=0.009）方面提示有统计学差异。

### 2.2 临床试验接受度及原因分析

在所有调查患者中，502例（93.3%）患者表示愿意参加或推荐亲友参加临床试验，490例（91.1%）患者表示愿意贡献剩余血液/组织标本用于研究。与未参加过试验的患者相比，参加过试验患者的临床试验接受度有所提高（90.8% *vs* 96.6%, *P*=0.008），但两者对贡献剩余血液/组织标本用于研究的意愿没有统计学差异（90.2% *vs* 90.6%, *P*=0.652）（图 1）。

**1:**
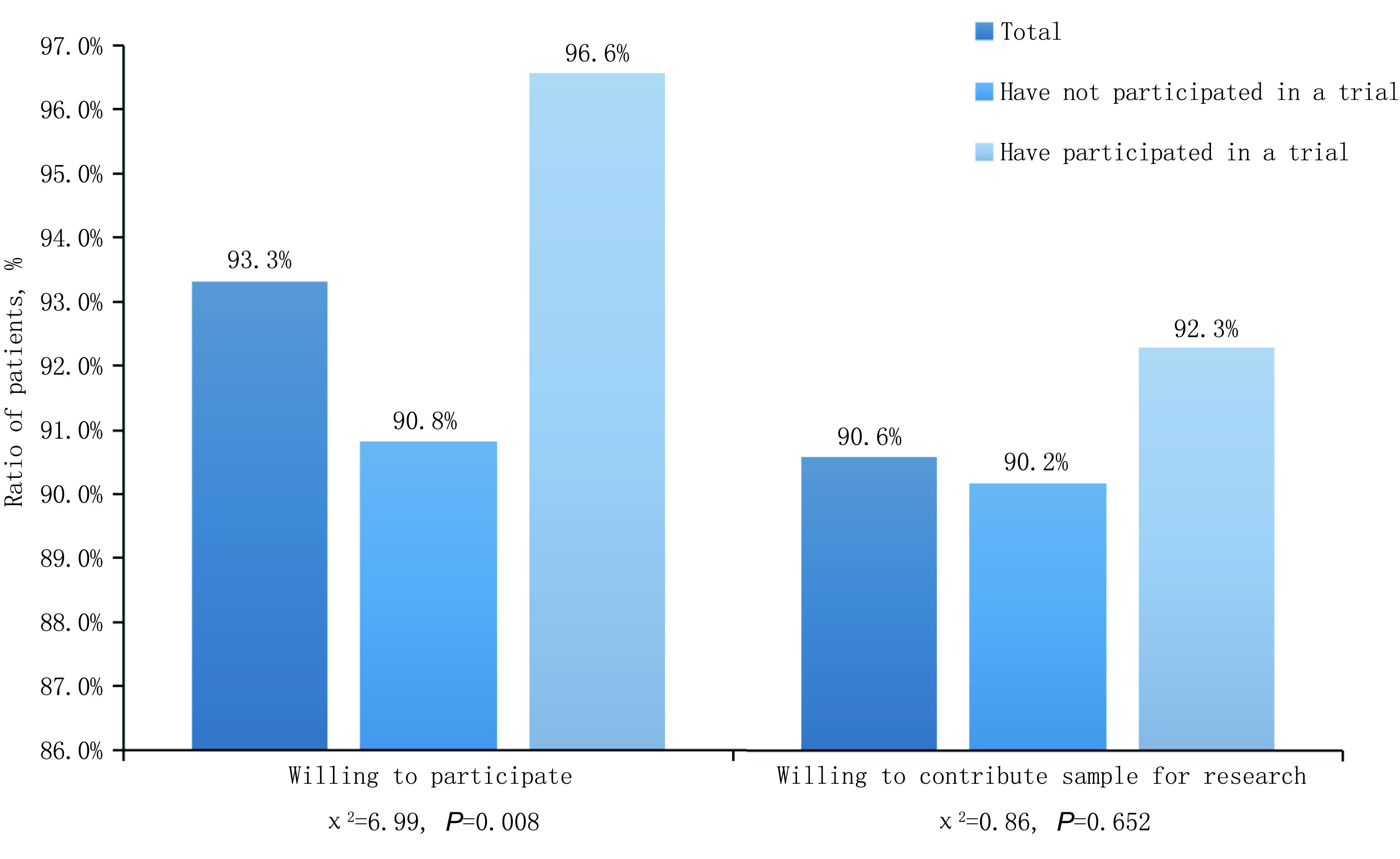
肿瘤患者对于临床试验的接受度情况 Acceptance of clinical trials in cancer patients

分析愿意参加或推荐亲友参加临床试验的相关原因，结果显示“得到最佳治疗效果”是几乎所有患者（99.6%）的主要原因之一。对于未参加过试验的患者，次要原因分别为“主治医生建议参加”（43.7%）和“可减轻经济负担”（38.6%）；参加过试验患者的次要原因分别为“可减轻经济负担”（56.0%）和“主治医生建议参加”（45.8%）。其他原因还包括“所患疾病没有有效治疗方法”、“为医学研究及今后患者尽己之力”和“得到更好的医疗服务”（图 2A）。

**2:**
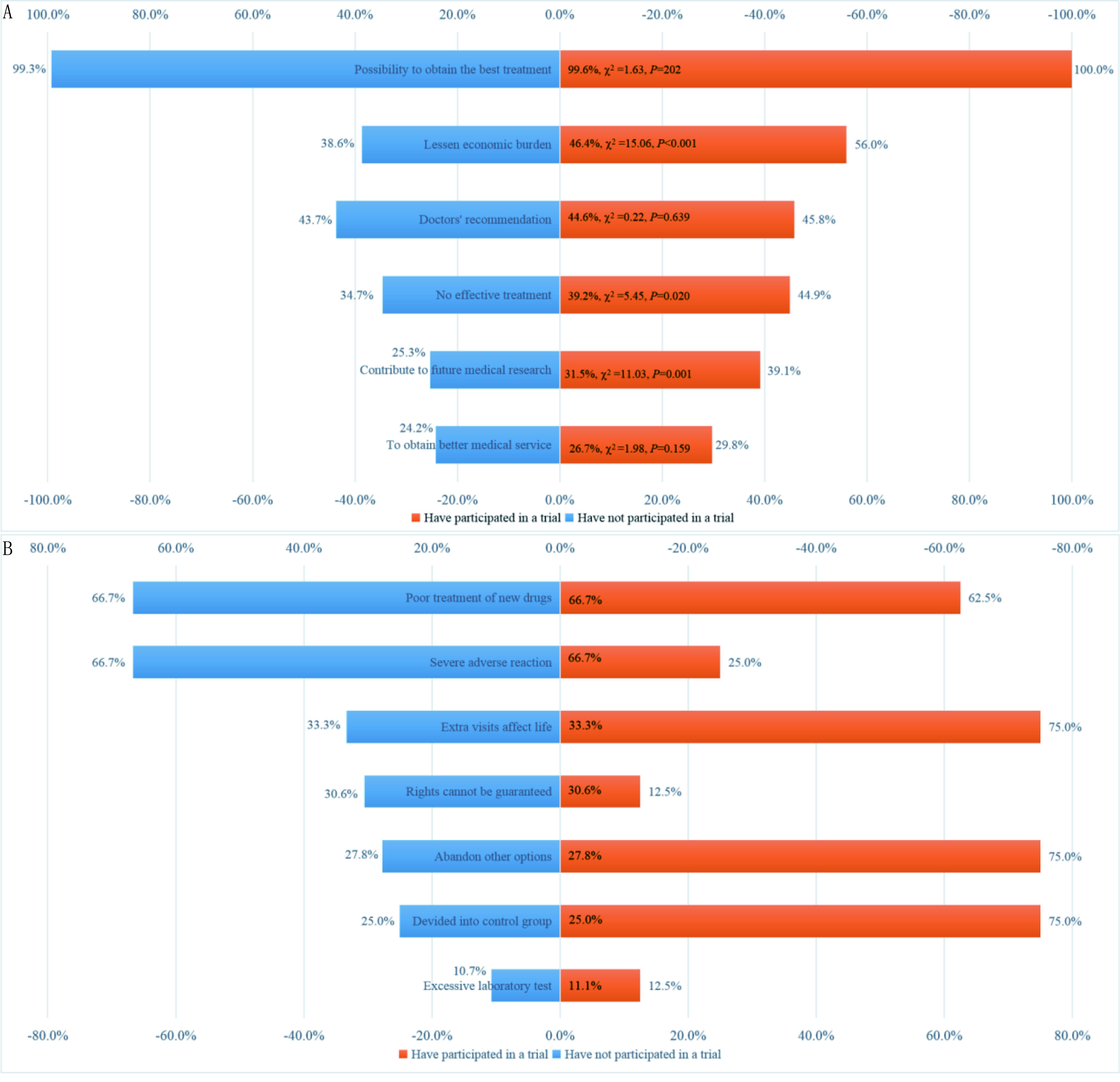
肿瘤患者愿意/不愿意参加临床试验的原因分析。A：愿意参加临床试验的原因；B：不愿意参加临床试验的原因。 Related reasons why cancer patients are willing/unwilling to participate in clinical trials. A: Reasons why patients are willing to participate in clinical trials; B: Reasons why patients are unwilling to participate in clinical trials.

**1:** 参与问卷调查患者的基本人口学信息[*n* (%)] Basic demographic information of patients participating in the questionnaire [*n* (%)]

Variable	Total (*n*=538)	Have participated in a clinical trial (*n*=233)	Have not participated in a clinical trial (*n*=305)	Statistics	*P*
Age (Mean±SD)	53.5±12.6	53.1±11.7	53.8±13.3	0.62	0.538
Gender				1.44	0.230
Male	27 (51.1)	126 (54.1)	149 (48.9)		
Educational Level^a^				2.82	0.244
Junior high and below	193 (36.0)	84 (36.1)	109 (36.0)		
Senior high	232 (43.3)	108 (46.4)	124 (40.9)		
University and above	111 (20.7)	41 (17.6)	70 (23.1)		
Occupation^b^				7.38	0.016
Full-time employees	179 (34.6)	66 (29.2)	113 (38.8)		
Peasantry	92 (17.8)	43 (19.0)	49 (16.8)		
Unemployed	115 (22.2)	49 (21.7)	66 (22.7)		
Retired	131 (25.3)	68 (30.1)	63 (21.7)		
Relatives and friends in medical industry				0.30	0.584
Yes	194 (36.1)	81 (34.8)	113 (37.1)		
Attention to health program				17.7	0.001
Often	142 (26.4)	60 (25.8)	82 (26.9)		
Usually	171 (31.8)	93 (39.9)	78 (25.6)		
Rarely	161 (29.9)	63 (27.0)	98 (32.1)		
Never	64 (11.9)	17 (7.3)	47 (15.4)		
Impression of medical staff				6.86	0.009
Great	331 (61.5)	158 (67.8)	173 (56.7)		
Good/General/Bad	207 (38.5)	75 (32.2)	132 (43.3)		
^a^*n*=536; ^b^*n*=517					

患者不愿参加或推荐亲友参加试验的主要原因也涉及多个方面。未参加过试验患者的主要顾虑包括“新药疗效不佳”（66.7%）和“严重不良反应”（66.7%），其次为“访视过多”（33.3%）、“自身权益无法保障”（30.6%），“不愿放弃其他治疗选择”（27.8%）。对于参加过试验的患者，主要顾虑包括“额外访视影响生活”（75.0%）、“放弃其他治疗选择”（75.0%）以及“分到对照组”（75.0%），次要顾虑包括“新药/新技术疗效不佳”（62.5%）和“严重不良反应”（25.0%）。“自身权益无法保障”和“化验检查过多”也是部分患者的顾虑（图 2B）。

### 2.3 主治医生对患者接受度的影响

对未参加过试验的305名患者，进一步调查主治医生建议对其决策的影响。研究发现，最初表达有了解和参加临床试验需求的患者比例分别为55.7%和77.4%，如果主治医生认为患者适合参加，此时表达有了解和参加需求的患者比例分别为98.0%和91.1%（图 3A）。对参加过试验的233例患者，进一步调查他们作出参与决策的关键人物发现，87.6%患者认为医生建议对决策起到关键作用，其他为自己决定（8.1%）、亲友建议（3.0%）和病友建议（1.3%）（图 3B）。

**3:**
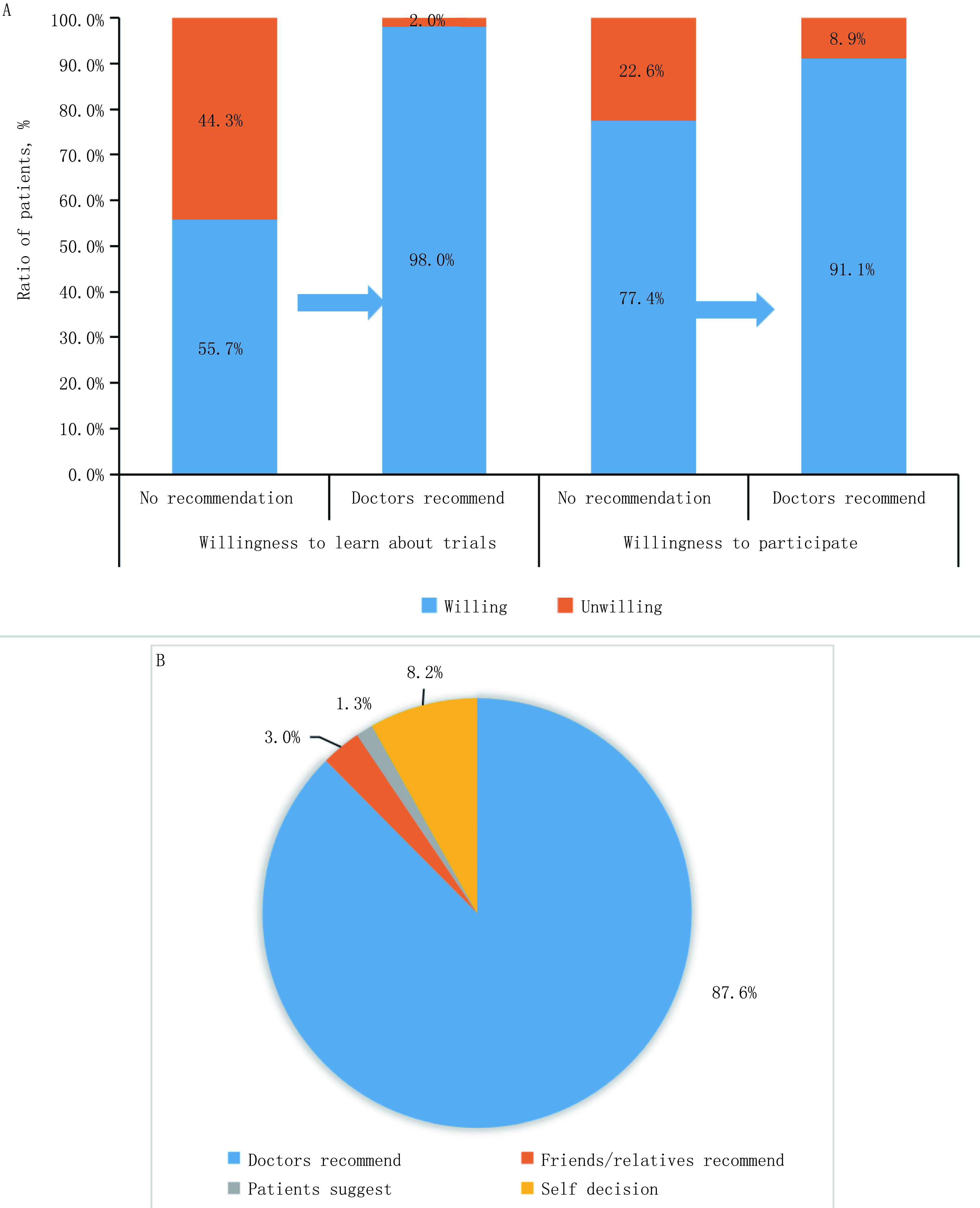
主治医生对肿瘤患者临床试验接受度的影响。A：未参加过临床试验的患者了解/参加临床试验的意愿；B：参加过临床试验的患者作出参与决策的关键因素。 Effect of attending doctors on the acceptance of clinical trials in cancer patients. A: Willingness-to-participate among patients who have not participated in a trial; B: Key factors in decision-making for patients who have participated in a trial.

### 2.4 临床试验的信息获取倾向

在希望获取的临床试验信息内容方面，未参加过和参加过试验的两组患者选择倾向较为一致，依次为“负责试验的医院和医生”（99.7% *vs* 99.6%, *P*=0.856）、“可能的治疗效果”（76.2% *vs* 74.9%, *P*=0.733）、“针对的疾病类型”（59.1% *vs* 59.7%, *P*=0.875）、“可能的风险”（53.7% *vs* 52.8%, *P*=0.841）和“是否免费”（39.6% *vs* 51.1%, *P*=0.008）。此外，还有“研究目的和性质”、“权益保障”、“研究申办方”、“研究阶段”、“医疗服务”及“研究的科研价值”（图 4）。

**4:**
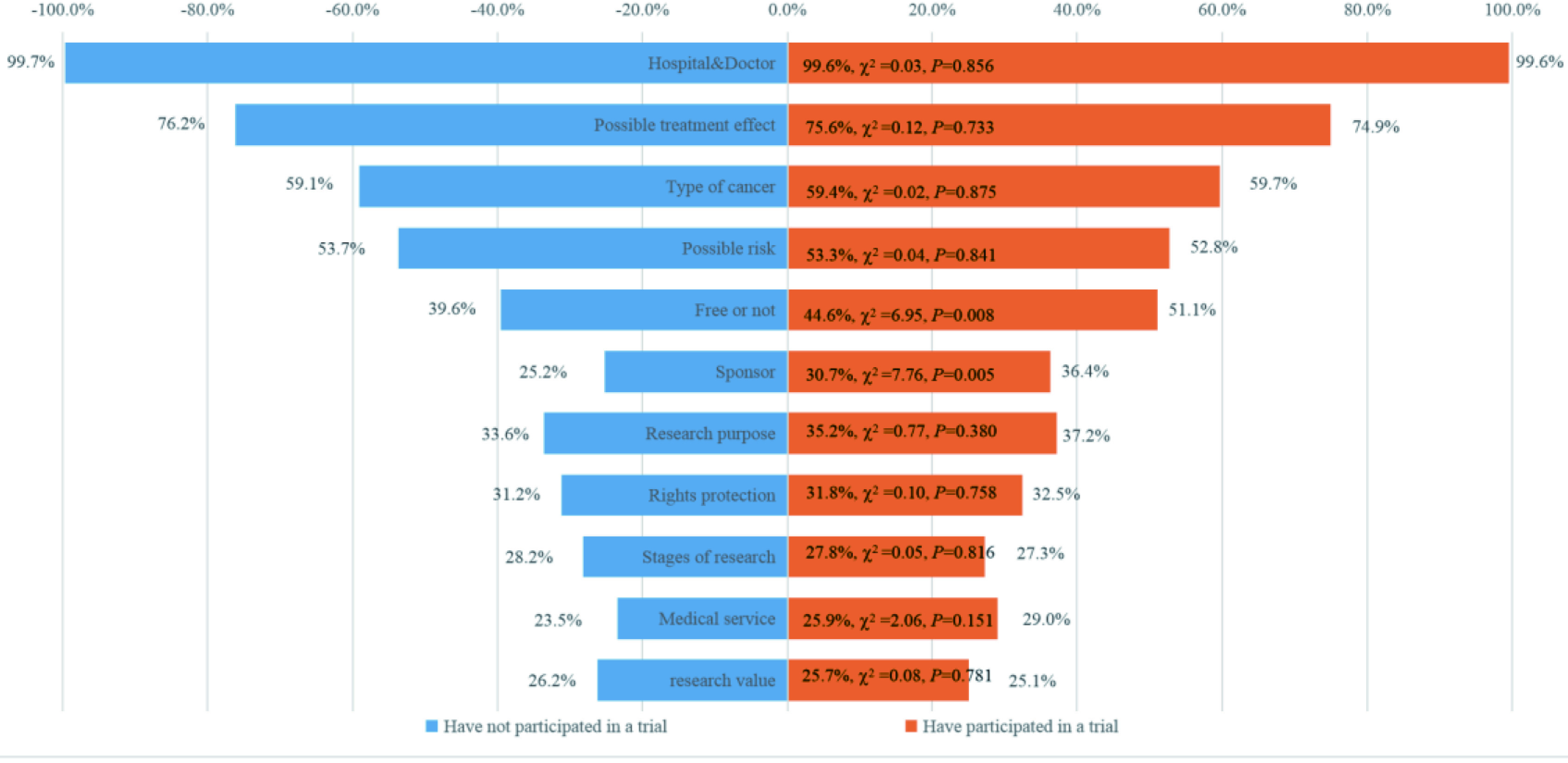
肿瘤患者希望获取的临床试验信息 Information of clinical trials that cancer patients expect to obtain

针对临床试验发起机构，多数患者倾向选择医院（66.6% *vs* 75.1%, *P*=0.032），其次为国内医药企业（35.7% *vs* 51.5%, *P*=0.0002）、国外企业（31.1% *vs* 38.2%, *P*=0.088）和学术研究机构（31.5% *vs* 31.8%, *P*=0.944）。在临床试验获取途径方面，几乎所有患者希望通过临床医生推荐（99.3% *vs* 100%, *P*=0.217），其次为医院宣传（39.0% *vs* 53.9%, *P*=0.001）和病友推荐（22.0% *vs* 37.9%, *P* < 0.000, 1）。新闻媒体、互联网和宣传单不是患者期望的信息获取途径（图 5）。

**5:**
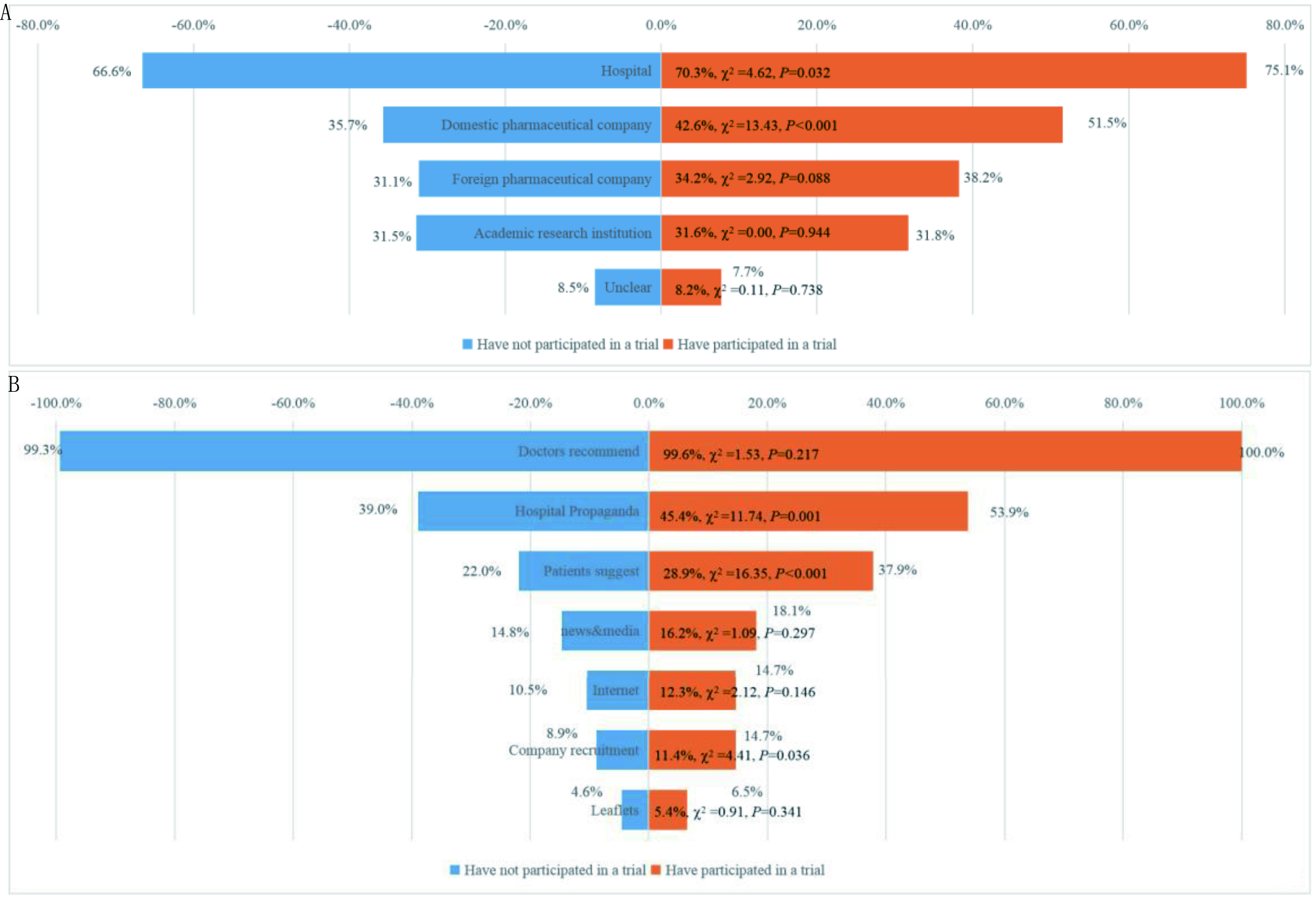
肿瘤患者临床试验信息获取渠道及参加倾向。A：患者倾向参加的临床试验发起机构；B：患者倾向的临床试验信息获取途径。 Access to clinical trial information and participation tendency of cancer patients. A: Sponsors of clinical trials that patients tend to choose; B: Access to clinical trial information for patients.

## 3 讨论

本研究通过问卷调查发现，肿瘤患者临床试验接受度普遍较高（93.3%），尤其是参加过临床试验的患者，绝大部分患者愿意贡献剩余血液/组织标本用于研究。影响患者临床试验接受度的因素涉及多个方面，试验药物的治疗效果以及医生建议是其中最重要的因素，几乎所有患者选择通过临床医生推荐进入试验，并希望获取负责试验的医院和医生信息。

不管与我国既往研究（40.9%-53.0%）^[5-8]^还是国外研究（56.7%-88.0%）^[9, 10]^比较，本研究得到的肿瘤患者临床试验接受度相对较高。可能与以下几方面原因有关：①随着临床试验在中国的广泛开展，大众和患者对临床试验的认知有所提高，继而接受度提高^[8]^；②我国公立医院的优质资源相对集中^[11]^，患者对于国家级医院的信任度和依从性都可能更好，这可能也是患者愿意贡献剩余标本/血液用于科学研究的原因；③患者参与试验的原因分析显示，减轻经济负担和更好的医疗服务是影响参与度的主要因素之一。

研究也显示与未参加过试验的患者相比，参加过试验患者的接受度更高（90.8% *vs* 96.6%）。这可能与以下因素有关：①参加过试验患者经历过知情同意告知过程，对临床试验有更准确和深刻的认知^[8]^；②参加过试验患者体验了中国医学科学院肿瘤医院专职研究团队为试验患者提供的全免费医疗服务，对参加试验的满意度较高。

研究多项数据提示主治医生对提高肿瘤患者临床试验接受度至关重要，包括主治医生推荐对未参加过试验人群的接受度影响和参加过试验人群的决策作用等。既往国内外相关研究也表明，医护人员尤其是主治医生的推荐是患者决定参加临床试验的关键原因，与本研究的结果类似^[12-15]^。因此，充分发挥主治医生的作用无疑对提高我国肿瘤患者临床试验接受度有重要意义。2019年卫健委科教司发布“重大新药创制”专项建设示范性药物临床评价技术平台，积极提倡采取措施建立有效途径向全院所有临床医生及时发布试验信息，激励和促进临床医生对临床试验研究的参与度，加强研究型医生的培养^[16]^。

此外，从影响患者参加临床试验的相关原因分析和希望了解的临床试验信息为我们未来的工作方向给出诸多提示，尤其是在伦理审查、知情同意和临床试验管理方面。（1）治疗效果始终是患者最关切的问题，把握临床前研究证据的科学性和充分性是伦理审核的重点，也是受试者权益保护的重要内容。（2）进行充分的知情告知，包括研究可能的风险受益、不良反应、是否免费、试验分组、可能涉及的访视等十分有必要。（3）加强试验过程中对患者不良事件的关注、管理和应对也是提升患者临床试验接受度的有效措施。同时，针对临床试验基本知识加强宣传教育，减少患者不必要的顾虑，亦可提升患者的参与意愿。

2013年，国家药品监督管理局建立了药物临床试验登记与信息公示平台^[17]^，要求所有在我国开展的以注册为目的药物临床试验必须在该平台登记，这极大促进了我国临床试验信息的权威性和管理的透明性。但由于多数患者不知道检索平台的存在、平台的检索不支持多条件筛选检索等功能，限制了患者从中获取甄别适宜临床试验项目的可能。目前，我国临床试验的发布途径主要还是针对单个项目张贴招募海报，部分通过商业招募网站、医院官网或公告栏公布等^[18]^，缺乏权威可视化的公布途径。

本研究创新性地进行了参加过试验患者和未参加过试验患者在临床试验参与度及相关原因方面进行的比较分析，同时深入讨论主治医生、患者对临床试验内容和获取途径方面的倾向性。但研究存在以下局限性。首先，本调查为单中心研究，缺乏全国代表性；其次，受限于实际调查工作开展环境，本研究采取了方便抽样，而非随机抽样；另外，尽管本研究的质量评价部分显示，纳入患者信息的可靠性较高，但调查整体人群参与度较低，依从性较为有限。以上因素均可能影响本研究结果的外推性。

综上所述，我国肿瘤患者临床试验接受度普遍较高，尤其是参加过临床试验的患者。充分发挥主治医生的作用，加强临床试验教育宣传对提高我国肿瘤患者临床试验接受度有重要意义。建立权威的临床试验可视化平台是未来工作的重要方向。
